# Swine influenza-modified pulmonary microbiota

**DOI:** 10.3389/fcimb.2025.1634469

**Published:** 2025-09-08

**Authors:** Javier Arranz-Herrero, Sara Izpura-Luis, Jesus Presa, Paloma Reche, Paloma Encinas, Taeyong Kwon, Sergio Rius-Rocabert, Vicent Tur-Planells, Juan Luis Tejerina, Jordi Ochando, César B. Gutiérrez-Martín, Eric Bortz, Adolfo Garcia-Sastre, Juergen A. Richt, Maria Montoya, Gustavo del Real, Estanislao Nistal-Villan

**Affiliations:** ^1^ Microbiology Section, Departamento de Ciencias Farmacéuticas y de la Salud, Facultad de Farmacia, Universidad San Pablo-CEU, CEU Universities, Madrid, Spain; ^2^ Transplant Immunology Unit, National Center of Microbiology, Instituto de Salud Carlos III, Madrid, Spain; ^3^ Institute of Applied Molecular Medicine (IMMA), Department of Basic Medical Sciences, Facultad de Medicina, Instituto de Medicina Molecular Aplicada-Nemesio Díez (IMMA-ND), Universidad San Pablo-CEU, CEU Universities, Madrid, Spain; ^4^ Independent Researcher, Madrid, Spain; ^5^ Department of Biotechnology, National Institute of Agricultural and Food Research and Technology (INIA, CSIC), Madrid, Spain; ^6^ Department of Diagnostic Medicine/Pathobiology, College of Veterinary Medicine, Kansas State University, Manhattan, KS, United States; ^7^ Department of Microbiology, Icahn School of Medicine at Mount Sinai, New York, NY, United States; ^8^ Department of Oncological Sciences, Icahn School of Medicine at Mount Sinai, New York, NY, United States; ^9^ Department of Animal Health, Faculty of Veterinary, Universidad de León, León, Spain; ^10^ Department of Biological Sciences, University of Alaska Anchorage, Anchorage, AK, United States; ^11^ Global Health and Emerging Pathogens Institute, Icahn School of Medicine at Mount Sinai, New York, NY, United States; ^12^ Department of Medicine, Division of Infectious Diseases, Icahn School of Medicine at Mount Sinai, New York, NY, United States; ^13^ Department of Pathology, Molecular and Cell-Based Medicine, Icahn School of Medicine at Mount Sinai, New York, NY, United States; ^14^ The Tisch Cancer Institute, Icahn School of Medicine at Mount Sinai, New York, NY, United States; ^15^ The Icahn Genomics Institute, Icahn School of Medicine at Mount Sinai, New York, NY, United States; ^16^ Viral Immunology Lab, Molecular Biomedicine Department, BICS Unit, Margarita Salas Center for Biological Research (CIB-CSIC), Madrid, Spain

**Keywords:** coinfection, influenza virus, lung, sequencing, respiratory microbiome, pigs, swine, Oxford Nanopore

## Abstract

Influenza A virus (IAV) remains a major health concern in both humans and animals, with pigs serving as key reservoirs for generating novel reassortant viruses with pandemic potential. Respiratory microbiome alterations during infection may facilitate secondary bacterial complications. This study investigates the lung microbiota of pigs naturally infected with IAV across different regions in Spain, using Oxford Nanopore Technologies (ONT) long-read 16S rRNA sequencing to characterize associated bacterial communities. Our results show a higher bacterial genus diversity in IAV-infected animals compared to healthy controls, with significant differences in both presence and relative abundance of bacterial taxa. Infected lungs exhibited increased proportions of potential pathogens, particularly *Glaesserella* spp., detected in approximately 60% of infected samples, often as the dominant genus. Other pathogenic genera, including *Pasteurella*, *Staphylococcus*, *Mycoplasma*, and *Fusobacterium*, were also strongly associated with infection. Clustering analyses revealed distinct microbial profiles that clearly separated infected from non-infected animals, identifying specific bacterial signatures predictive of infection status. These findings suggest that IAV infection significantly alters the pulmonary microbiota, potentially creating a permissive environment for secondary bacterial infections. This study underscores the relevance of microbiota shifts during IAV infection in swine and highlights the importance of understanding microbial dynamics in respiratory disease progression. Additionally, we present a novel, rapid, and practical experimental pipeline based on ONT long-read sequencing to investigate the respiratory microbiota in swine infection models. This approach offers a valuable tool for future research and potential diagnostic applications in both veterinary and human medicine.

## Introduction

Influenza viruses are a significant concern in both veterinary and public health, with swine serving as important reservoirs for various Influenza A virus subtypes. The potential for generating novel reassortant viruses and subsequent zoonotic transmission to humans underscores the importance of understanding the dynamics of influenza in swine populations (Zell et al., 2013). Moreover, recent pandemics, such as the H1N1 in 2009, with swine being the mixing vessel for the resulting reassortant viruses with four different original Influenza viruses from human, bat and pigs (Kingsford et al., 2009), highlights the need for comprehensive surveillance and research in this area.

Currently there is great interest in unravelling the role of microbiota in viral infections, given that it could be a potential therapeutic target or an important prognostic marker. The term “microbiota” refers to the collection of microorganisms, primarily bacteria, that inhabit a specific environment in the body. Efforts are currently being made in order to develop future therapeutic approaches in the microbiome area (Jin et al., 2022). Bacterial microbiota is present in many organs, including the respiratory tract, and it appears to play a role in the pathogenesis or progression of several diseases. In the lungs, alterations in microbial composition have been associated with chronic obstructive pulmonary disease (COPD), asthma, cystic fibrosis, and respiratory infections, where shifts in microbial diversity or abundance often correlate with disease severity or exacerbations (Garrett, 2015; Budden et al., 2019; Wilde et al., 2024). Furthermore, the lung microbiota has drawn considerable attention due to the intricate immunological tolerance mechanisms that are necessary to preserve a balanced and symbiotic interaction between resident microbes and the host in the respiratory environment.

The respiratory microbiota plays a fundamental role as a potential source of influenza-associated secondary opportunistic bacterial infections (Li et al., 2021). While influenza-induced changes in the intestinal microbiota are well documented in animal models, direct effects on the respiratory microbiota remain understudied (Li et al., 2021). Only some investigations analyzing sputum samples in humans have also reported significant alterations in respiratory microbial communities following influenza infection (Zhou et al., 2023a). Unlike other internal organs, the lungs are exposed to both external and internal factors that create a highly dynamic microbial environment (Dickson et al., 2015b; Dickson et al., 2016). Although lungs were once thought to be sterile (Dickson et al., 2016; Whiteside et al., 2021), the pulmonary microbiome of swine represents a complex ecosystem comprising diverse bacterial communities. These microbial communities may play a critical role in the development of secondary infections, which are directly associated with increased morbidity and mortality following Influenza infection (Li et al., 2021).

Despite its potential importance, our understanding of the porcine respiratory microbiome is still limited, presenting an exciting opportunity for further investigation. Traditionally, the identification of pulmonary microbiota in swine has relied on culture-based methods, which are inherently limited by their inability to capture the full breadth of microbial diversity (Tunney et al., 2013). Although these methods have identified the main secondary pathogens associated with pneumonia in swine [*Streptococcus suis*, *Haemophilus parasuis*, *Pasteurella multocida*, among others (Zhao et al., 2021)], most of the bacteria present in the lungs and the respiratory environment are non-cultivable using conventional techniques and require enrichment factors, leading to a significant loss of information about their ecological role during cultivation. To overcome this limitation, alternative approaches such as high-throughput sequencing of the 16S rRNA gene have emerged as powerful tools for characterizing microbial communities (Li et al., 2021).

**Figure 1:**
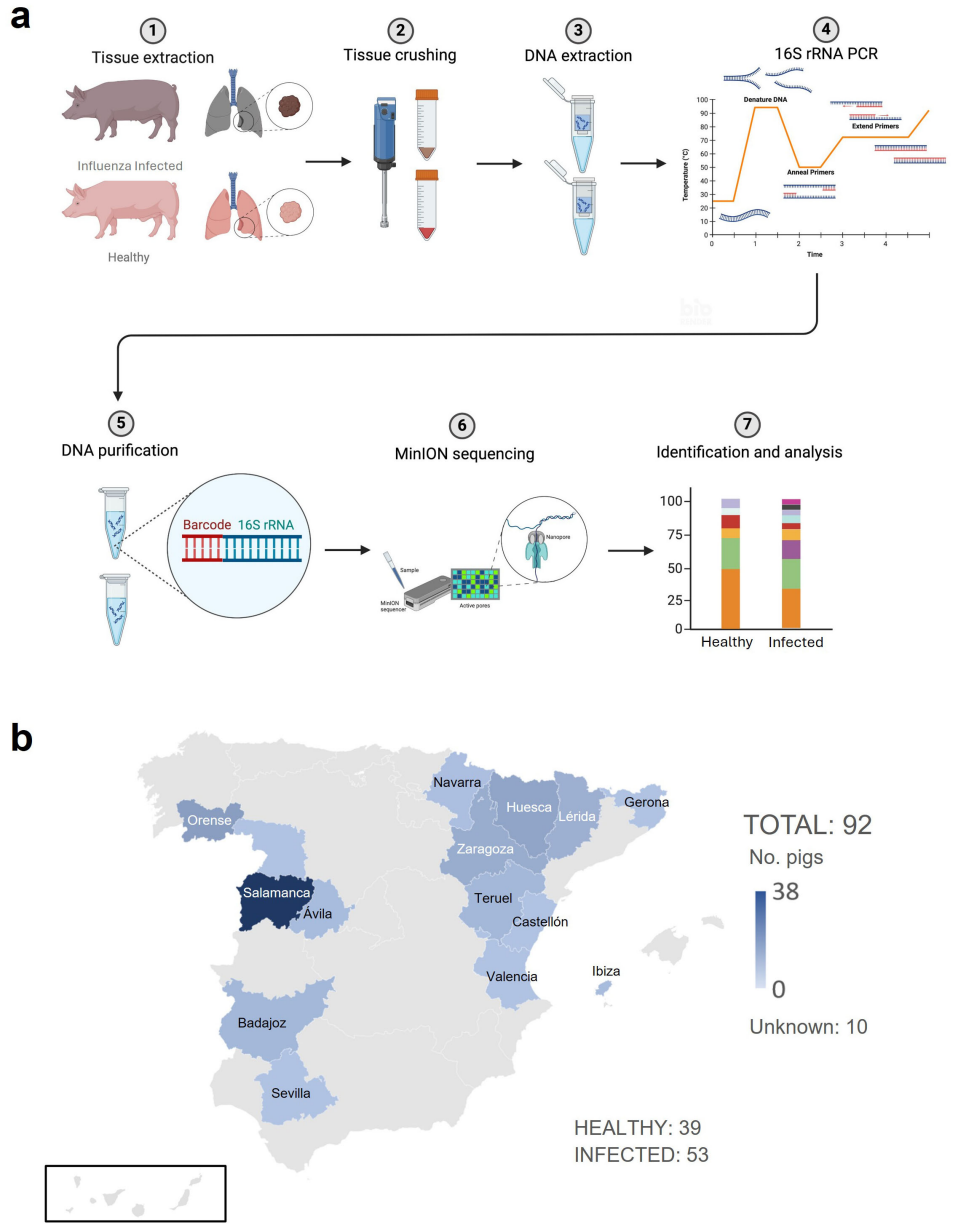
Graphical abstract. **(a)** Schematic and Graphical Representation of Experimental Methodology. A piece of lung from all infected pigs was extracted, embedded in PBS (Phosphate Buffered Saline) (1) and crushed (2) using a TissueLyser. Then, DNA was extracted (3) using Qiagen extraction kit and amplified (4) with barcoded 16S primers from Oxford Nanopore Technologies. Purification of the DNA (5) was performed using magnetic beads for the library preparation and sequencing (6). Finally, data were analyzed (7). **(b)** Number and origin of the pigs analyzed. 92 samples were processed and analyzed: 53 were Influenza-infected and 39 were (non-infected) - healthy animals. Figure created with BioRender.com.

In this study, we employed Oxford Nanopore sequencing technology (ONT) to investigate the pulmonary microbiome of pigs infected with Influenza virus from different locations in Spain with a targeted Next Generation Sequencing (NGS) metagenomic approach, by generating long-read 16S rRNA sequences (**Figure 1** – Graphical abstract). This analysis provides a more comprehensive assessment of bacterial diversity and composition in the context of Influenza infection and pathogenesis, offering insights into potential frameworks for disease understanding.

## Materials and methods

### Sample collection

Clinical samples (lung, trachea, and nasal exudates) from healthy (39) and influenza infected pigs (53) with respiratory symptoms were provided by farm veterinarians from main pig-producing regions of Spain. Samples were stored at -20°C and sent in ice to maintain the cold chain. A preliminary diagnostic test of Influenza A-specific RT-PCR was performed at EXOPOL S.L. (San Mateo de Gállego, Spain). Positive samples were confirmed by RT-PCR in our laboratory. Non infected samples (PCR negative) were named as healthy.

### Pulmonary tissue processing and nucleic acids extraction

A tissue fragment (25 mg only containing soft tissue from a lobe, no trachea or bronchioles) was embedded in 5 mL of PBS and crushed with an ultra-turrax until no big and detectable fragments were visible (until the mixture was homogeneous). Then, DNA extraction was performed using QIAAMP DNA MINI KIT (Qiagen) according to the manufacturer’s instructions for tissues. Specifically, 80 µL of the homogenate was mixed with 100 µL of buffer ATL and proteinase K and incubated overnight at 56°C. RNase A (100 mg/mL) was added and incubated for 2 minutes at room temperature to remove RNA. Then, 200 µL of buffer AL was added and incubated for 10 minutes at 70°C, followed by the addition of 200 µL of 100% ethanol. The lysate was loaded onto spin columns and centrifuged at 6000 g. Wash steps with buffers AW1 and AW2 were performed according to the protocol, including a high-speed spin (20,000 g) and a final drying step. DNA was eluted in two rounds using 100 µL of DNase-free AE buffer, with 1–5 min incubation before each spin to improve yield.

### 16S/18S ratio obtention

Extracted DNA from healthy and infected samples were amplified using quantitative PCR technique using SYBR Premix Ex Taq (Takara Kusatsu, Shiga, Japan) following manufacturer instructions in a 7900HT fast real-time PCR system (Thermo Scientific, Waltham, MA, USA) using the primers needed to quantify the expression of the genes under analysis, designed using an in-house program (16S_Fw: TGTCGTGAGATGTTGGG, 16S_Rv: CGATTCCAGCTTCATGT; 18S_Fw: CCAAGATCCAACTACGAGCTT, 18S_Rv: GGCCCTGTAATTGGAATGAGTC). Thermal cycling conditions were as follows: an initial incubation of 2 minutes at 50°C, followed by a denaturation step of 10 minutes at 95°C. Amplification was then performed for 40 cycles of 15 seconds at 95°C, 15 seconds at 60°C, and 15 seconds at 72°C. A melting curve analysis was included at the end to confirm specificity of amplification. Samples with undetermined 16S amplification after 40 cycles were considered below the detection limit. Then, the ΔΔCt (2^(Ct16S – Ct18S)^) for 16S/18S was calculated, and normalized with the lowest value over the detection limit. The threshold for the limit of detection (LOD) was set based on the theoretical maximum Ct value of 40 for 16S amplification, in combination with the average Ct value observed for 18S across samples. This resulted in a LOD ratio of approximately 15, below which samples were considered under the detection limit and excluded from statistical analysis.

This ratio serves as an independent proxy of bacterial load relative to host tissue, allowing comparison of bacterial abundance per unit of lung tissue between healthy and infected samples, independently of sequencing depth or amplification biases.

### 16S rRNA PCR amplification and purification

10 ng of DNA were used to amplify the 16S rRNA gene of the bacteria present in the sample using the 16S Barcoding kit (SQK-16S024) from Oxford Nanopore Technologies (ONT), according to the manufacturer’s instructions. The Taq Polymerase used was LongAmp Hot Start Taq 2X Master Mix (NEB, M0533S), as indicated in the protocol. After amplification, DNA was purified using HighPrep beads (Magbio - AC-60005) and eluted in 10 µl of 10 mM Tris-HCl pH 8.0 with 50 mM NaCl.

### MinION sequencing of the 16S rRNA gene and bacteria identification

50–100 fmoles of purified DNA were pooled in 10 μL of Elution Buffer and sequenced using the MinION Mk1b ONT. For priming and loading the FlowCells, the Flow Cell Priming Kit (EXP-FLP002) was used according to the manufacturer’s instructions. Briefly, the Rapid Adapter (RAP) was added to the purified sample and incubated for 5 minutes. During this time, flow cells were primed by mixing Flush Tether (FLT) with Flush Buffer (FB), and loading 800 μL of this mix into the flow cell after air removal. The final sequencing mix was prepared by combining the RAP-treated sample with Sequencing Buffer (SQB), Loading Beads (LB), and water to a final volume of 75 μL. An additional 200 μL of the FLT/FB mix was loaded via the priming port, and the library was then added dropwise onto the SpotON port. All flow cells were checked before sequencing and run parameters were set equally for all runs during a maximum run limit of 72h. Basecalling was performed in real time using the MinKNOW software (v22.12.5), which integrates the Guppy basecaller under default settings. All sequencing runs were subjected to quality control, and samples with extremely low read counts (*e.g*., <100 high-quality reads) were excluded from further analysis.

### Data analysis

Bacterial/host (16S/18S) ratios were obtained by normalized relative copy number and compared with a non-parametrical Mann-Whitney test between samples over the detection limit. Normality of the data was assessed using multiple tests, including the Anderson–Darling, D’Agostino–Pearson, and Shapiro–Wilk tests, all of which indicated a significant deviation from normality. A p-value < 0.05 was considered statistically significant.

Epi2me desktop application (Oxford Nanopore Technologies) was used for identification and bacterial classification using the *metagenomics* workflow. This workflow performs taxonomic assignment of mixed samples and provides genus-level resolution based on single-read alignments using either Kraken2 or Minimap2. In our experiment, Kraken2 (v2.0.9-beta) (Wood et al., 2019) was as selected for the alignment of reads to Standard-8 database (incorporated in Kraken2 tool). This database includes 246,068 different species. All analyses were conducted using the web-based Epi2me interface under default settings. No command-line tools or R environments were used for this step.

An initial filter of length (200bp – 5000bp) was set during base-calling to discard both short and long reads as failed reads. Short reads may add noise to the analysis, and long reads were not targeted as the analysis focused on the complete barcoded 16s genes. The maximum statistical significance threshold (E_value_) was set as default [e=0.01], and minimum coverage was set at 30%. In this context, “coverage” refers to the proportion of each read that is aligned to a reference sequence in the Standard−8 database; reads must align over at least 30% of their length to be considered for taxonomic classification. These settings correspond to the default parameters of the Epi2me Metagenomics workflow and were not manually modified.

After obtaining the taxonomic and abundance of bacterial reads using Kraken2 in the Epi2me platform, the microbial composition of samples and groups was analyzed and plotted using R (v4.3.3). Alpha and beta diversity metrics were calculated using the *microbiomeStat* R package v0.2.1 (Zhou et al., 2022) applying the analysis to the full dataset without rarefaction in order to preserve the total read information. Specifically, alpha diversity was estimated using Chao1, ACE, Shannon, and Simpson indices, while beta diversity was assessed using Bray–Curtis distances and visualized through MDS (Multidimensional Scaling) plots. Statistical differences in alpha diversity indices and beta diversity distances between infected and healthy groups were assessed using the LinDA method implemented in the microbiomeStat R package, which fits linear models adjusted for potential confounders. Significance was determined based on p-values adjusted for multiple testing. In addition, a species accumulation curve (rarefaction curve) was generated using the *vegan* R package v2.7−7 (Oksanen et al., 2025) to explore the relationship between the number of samples and the number of detected species. As expected, the shape of the curve varied depending on the order of sample addition due to differences in richness among samples; a representative run showing a typical logarithmic shape was selected for illustration. Statistical differences in alpha and beta diversity between infected and healthy groups were assessed using the LinDA method implemented in the *microbiomeStat* R package, which fits linear models adjusted for potential confounders. Significance was determined based on p-values adjusted for multiple testing. To identify differentially abundant taxa between healthy and infected animals while accounting for the compositional and sparse nature of microbiome count data, we additionally applied the ANalysis of Composition of Microbiomes with Bias Correction (ANCOM-BC), implemented in the ANCOMBC R package v2.10.1 (McMurdie and Holmes, 2013; Lin and Peddada, 2020). This method was run at multiple taxonomic levels (phylum, class, order, family, genus), and results were used to complement the interpretation of taxonomic shifts between groups.

Then, a comparison between groups was performed by five different methods: 1) Wilcox rank sum test [*stats* R package (The R Core Team)], 2) biserial correlation function based on sensitivity/sensibility [*multipatt* function from *indicspecies R package* v1.7.12 (Cáceres et al., 2025)], 3) Pearson correlation based on presence [*multipatt R package* (De Cáceres and Legendre, 2009)], 4) LinDa method (*microbiomeStat* R package), and 5) Boruta [*Boruta* R package v7.0.0 (Kursa and Rudnicki, 2010)]. The significant bacteria from all methods were further selected for the generation of a prediction model using a Flexible Discriminant Analysis (FDA) using the *mda* R package v0.7−10 (Hastie et al., 2024). All methods were compared based on their discrimination abilities and the *Z* scores obtained.

## Results

**Figure 2:**
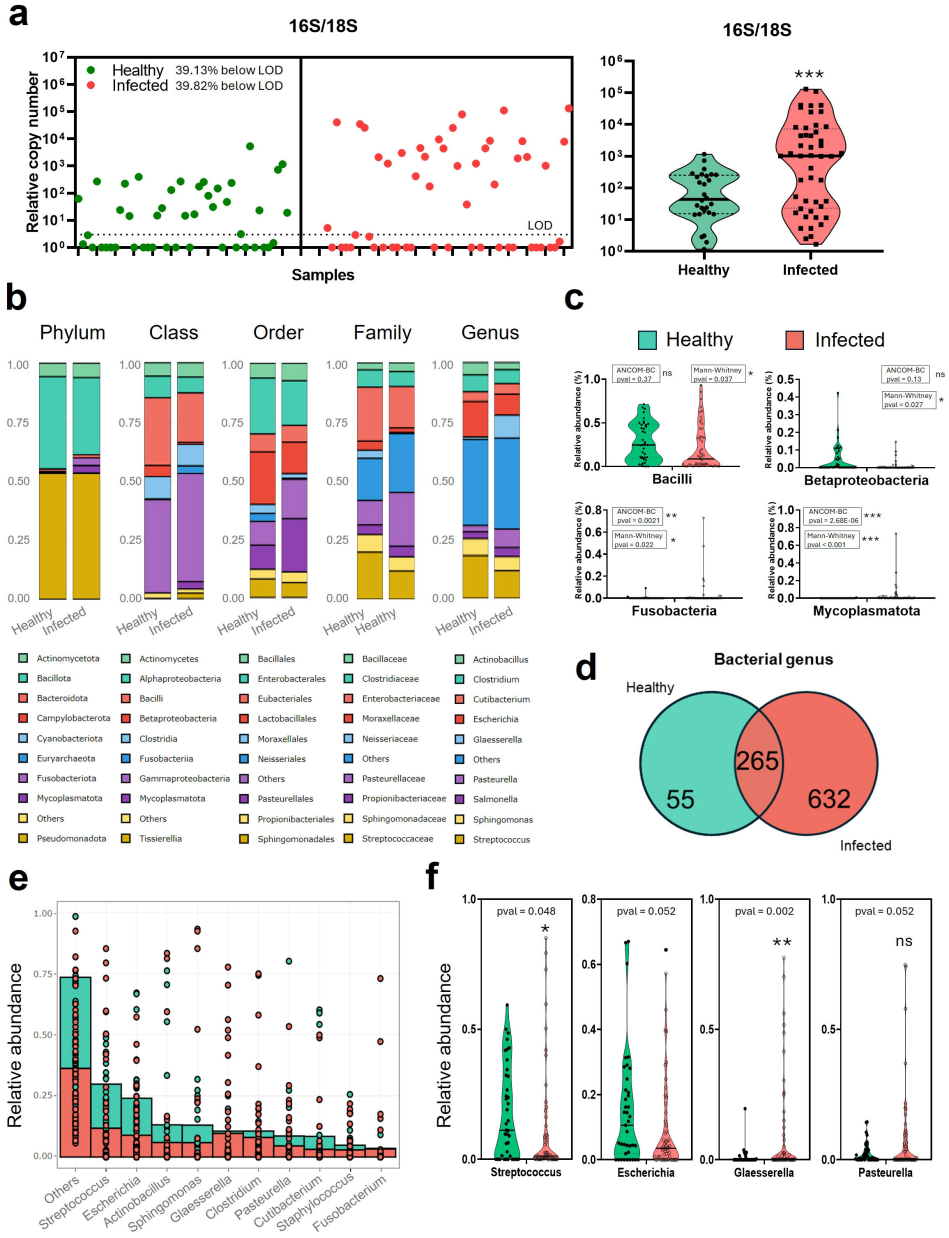
Relative bacterial abundance in the lungs of Influenza virus-infected and non-infected swine. **(a)** Individual 16S/18S gene ratio obtained by qPCR for 39 healthy (green) and 53 infected (red) DNA purified from lung necropsy samples. LOD: Limit of Detection **(b)** Stacked bar average relative abundance (%) of bacteria present in healthy (Influenza confirmed negative) pigs and Infected with influenza virus for phylum, class, order, family, and genus. **(c)** Violin plots of the main class groups classification of bacteria (Bacilli, Betaproteobacteria, Gammaproteobacteria, and Mollicutes) in Healthy vs Infected animals diagnosed with Influenza virus. **(d)** Venn diagram of bacteria in healthy or infected pigs at the genus level. **(e)** Individual bacterial stacked representation of genus relative abundance (%) ordered in terms of abundance. **(f)** Violin plots of relevant genus groups classification of bacteria (Streptococcus, Escherichia, Glaesserella, and Pasteurella) in healthy vs infected samples from Influenza virus-diagnosed swine. Statistical comparisons were performed using the Mann–Whitney U test and ANCOM-BC microbiome test. Significance levels are indicated as follows: · p ≈ 0.05, *p < 0.05; **p < 0.01; ***p < 0.001. ns, Non-signitican diferences.

Lung samples from healthy (39) and naturally infected pigs (53) were processed. Influenza infection was initially detected by RT-qPCR at EXOPOL S.L and further confirmed in our laboratory, with an average Ct value of 23.93 (± 3.52 SD) for positive samples. Then, total DNA was extracted from each sample and used to estimate the relative bacterial burden per tissue unit using qPCR targeting bacterial 16S and host 18S rRNA genes. The 16S/18S ratio was used as a proxy for bacterial abundance relative to host tissue. Analysis of these ratios revealed substantial variability across samples (**Figure 2a**-left). Notably, influenza-infected animals showed significantly increased bacterial loads compared to healthy controls (**Figure 2a**-right), with a median 16S/18S ratio of 787.7 (range: 1.116–1,163) versus 36.05 (range: 1.661–65,376) in healthy lungs (p = 0.0008, Mann Whitney test). Influenza infected samples had 21.85x median ratio compared to healthy samples.

### Taxonomic analysis and microbial diversity

All samples were sequenced using 16S Long-read Oxford Nanopore Technologies (ONT). MinKNOW Software was used to collect sequencing data, perform basecalling in real-time, and demultiplex the barcodes. Bacterial identification was performed using Epi2me, and relative abundance was calculated for all classification levels. 16S rRNA gene sequencing and comparisons were analyzed between the two groups (Infected vs Healthy).

A total of 1,519,055 reads were classified as “*Classification successful*”, meaning that a taxon could be assigned to those reads based on a valid NCBI Taxonomic ID (taxid). Unclassified reads were discarded as the results were not available or did not meet the criteria set at the start. Classification by Epi2me was assigned with Kraken2 after alignment of the read with the Standard-8 database (246,068 species). Taxonomic analysis revealed 40 bacteria phyla, 76 classes, 175 orders, 372 families, 953 genera, and 2361 species. Species level was not considered in the analysis as the low level of resolution might interfere with the results.

For alpha diversity analysis, bacterial community indices (Chao1 and ACE) were calculated for richness analysis, while Shannon and Simpson indices were calculated for diversity analysis. ACE and Chao1 indices indicated that the bacterial richness was not significantly different (p > 0.05) between groups, as determined by the LinDA method (**Supplementary Figure 1a**). Similarly, no significant differences (p > 0.05) were found in bacterial community diversity (Shannon and Simpson indices). However, rarefaction curves showed a higher number of observed genera in infected samples compared to healthy samples (**Supplementary Figure 1b**).

For beta diversity analysis, two-dimensional principal coordinates analysis (PCoA) based on Bray-Curtis (BC) distance using *MicrobiomeStat* R package (**Supplementary Figure 1c**), and three-dimensional BC dissimilarity using *Bracken* package from *Krakentools* were calculated (**Supplementary Figure 1d**). In this last analysis, other factors such as batch effect or the origin of the pigs were included. Beta diversity analysis revealed that the two groups were segregated into several different community clusters that are primarily dependent on the infection rather than the run or the region (**Supplementary Figures 1c, d**). Both beta diversity approaches showed consistent results, indicating that neither sequencing run nor geographical origin explained the sample clustering. Instead, infection status was the main factor driving the observed community differences. However, the cumulative percentage of explained variances of the first 2 and 3 dimensions represented only 17.19% and 23.37%, respectively (**Supplementary Figure 1e**).

### Microbial composition of lung microbiota

To illustrate the overall taxonomic composition, the nine most abundant groups at each taxonomic level (phylum, class, order, family, and genus) were represented based on mean relative abundance per group, and standard deviation (SD) values were calculated to describe group variability (**Figure 2b**). For phylum classification, Bacillota, Pseudomonadota, and Actinomycetota comprised the main bacterial community in both groups. However, other bacteria such as Fusobacteriota, and Mycoplasmatota, present in infected pigs, were low in healthy animals. In particular, infected pigs showed a non-significant decrease in the Bacillota phylum compared to healthy animals (32.7% [± 32.7% SD] vs 39.1% [± 25.1% SD], p-val_ANCOM -BC_ = 0.12). In contrast, infected pigs showed higher values for Fusobacteriota (3.27% [± 12.1% SD] vs 0.28% [± 1.46% SD], p-val_ANCOM -BC_ = 0.0021), and Mycoplasmatota (3.17% [± 10.8% SD] vs 0.04% [± 0.16% SD], p-val_ANCOM -BC_ = 2.68E-06) compared to healthy pigs. Within these phyla, in the class groups (**Figures 2b, c**), bacterial relative abundance in healthy pigs showed a higher, non-significant percentage of percentage of Bacilli (28.69% [± 21.9% SD] vs 20.99% [± 25.7% SD], p-val_ANCOM -BC_ = 0.37) and Betaproteobacteria (4.68% [± 0.08% SD] vs 0.80% [± 0.025% SD], p-val_ANCOM -BC_ = 0.13) compared to naturally infected animals. In the infected animals, there was a higher relative abundance of Gammaproteobacteria and Mycoplasmatota (39.48% [± 28.4% SD] vs 45.76% [± 36.5% SD], p-val_ANCOM -BC_ = 0.0042; and 0.028%, [± 0.1% SD] vs 3.12% [± 10.9% SD], p-val_ANCOM -BC_ = 1E-20, respectively). Deeper in the analysis, order classification showed a majority of Lactobacillales in healthy pigs (22.36% [± 20.9% SD] vs 13.46% [± 22.6% SD], p-val_ANCOM -BC_ = 0.93), but a higher prevalence of Pasteurellales in infected animals (10.28% [± 20.6% SD] vs 22.77% [± 29% SD], p-val_ANCOM -BC_ = 0.0047). Among them, the most abundant families were Streptococcaceae for healthy animals (19.93% [± 19.7% SD] vs 11.94% [± 21.7% SD], p-val_ANCOM -BC_ = 0.9), although Neisseriaceae was the most significant group for healthy animals (3.39% [± 7% SD] vs 0.4% [± 1.9% SD], p-val_ANCOM -BC_ = 0.0025); and Pasteurellaceae for infected ones (10.28% [± 20.6% SD]. vs 22.77% [± 29% SD], p-val_ANCOM -BC_ = 0.003).

Regarding genus abundance (**Figures 2d–f**), the number of unique and shared bacterial genera between the two groups was shown using Venn diagrams (**Figure 2d**). The total number of bacterial genera in the infected group was much higher than in the healthy group (498 versus 120), while 192 genera were shared between the two groups, suggesting that these shared genera might represent common inhabitants of lung microbiota. The prevalence of each bacterial genera identified in each group (healthy and infected) is represented in **Supplementary Figure 2a**. Prevalence is understood as the percentage of samples that identified the presence of the bacteria. Also, a table with the bacteria and each prevalence, as represented in the Venn diagram groups, is shown in **Supplementary Figure 2b**.

Analysis of the top 10 bacteria in the genus classification (**Figures 2e, f**) showed that *Streptococcus* is the most abundant bacteria for both infected (11.73% [± 21.48% SD], p-val_ANCOM-BC_ = 0.8) and healthy (18.05% [± 17.88% SD]) pigs. However, significant differences were found for *Glaesserella* and *Pasteurella* (**Figures 2d, e**), which were more abundant in infected animals (0.93% [± 3.22% SD] vs 9.6% [± 19.16% SD], p-val_ANCOM -BC_ = 3.1E-06 for *Glaesserella*; and 2.6% [± 3.9% SD] vs 7.97% [± 16.91% SD], p-val_ANCOM -BC_ = 0.012 for *Pasteurella*). In healthy animals, the most predominant genera with relative abundances over 1%, apart from *Streptococcus*, were *Escherichia* (15.12% [± 17.71% SD]), *Sphingomonas* (7.22% [± 18.73% SD]), *Clostridium* (7.15% [± 16.54% SD]), *Actinobacillus* (5.31% [± 15.44% SD]) or *Cutibacterium* (4.08% [± 13.67% SD]), among others. While in the infected animals, after *Streptococcus*, the most predominant bacteria were *Glaesserella*, (9.64% [± 19.16% SD]), *Escherichia* (8.88% [± 13.26% SD]), *Pasteurella* (7.95% [± 16.91% SD]), *Sphingomonas* (5.93% [± 17.61% SD]), *Clostridium* (5.87% [± 18.75% SD]), *Cutibacterium* (4.5% [± 9.57% SD]) or *Salmonella* (3.83% [± 4.49% SD]), among others. Interestingly, bacteria such as *Staphylococcus*, *Mycoplasma*, *Haemophilus*, *Bacillus*, *Trueperella* or *Salmonella* were also higher in the infected group. Analysis of the relative abundance of genus level in the individual samples (**Supplementary Figure 3**) showed that in infected animals there is a higher number of samples where there is a major predominance of a single bacteria. This is particularly clear for the already mentioned genera *Glaesserella* and *Pasteurella.* In contrast, the number of samples with *Streptococcus* in the infected samples decreased considerably. Also, this individual analysis included clusterization by regions (**Supplementary Figure 3**), where samples from the same regions were not necessarily from the same farm. However, it can be observed that in healthy animals, samples from the same region show similar patterns, while in infected animals, the observed relative abundances are despair.

### Comparison of lung microbial communities between the two groups

We further analyzed the significant differences between bacterial abundances in both groups using the Wilcoxon rank sum test method. To confirm that the Wilcoxon rank-sum test was the most suitable for our data, we also evaluated five additional statistical methods for comparison (**Supplementary Figures 4** and **5**). These methods were compared using a flexible discriminant analysis (FDA) predictive model based on the significant bacteria of each model. Finally, the discrimination abilities of each model after FDA and the *Z* score for each model validated the Wilcoxon test as the most suitable for these data.

**Figure 3:**
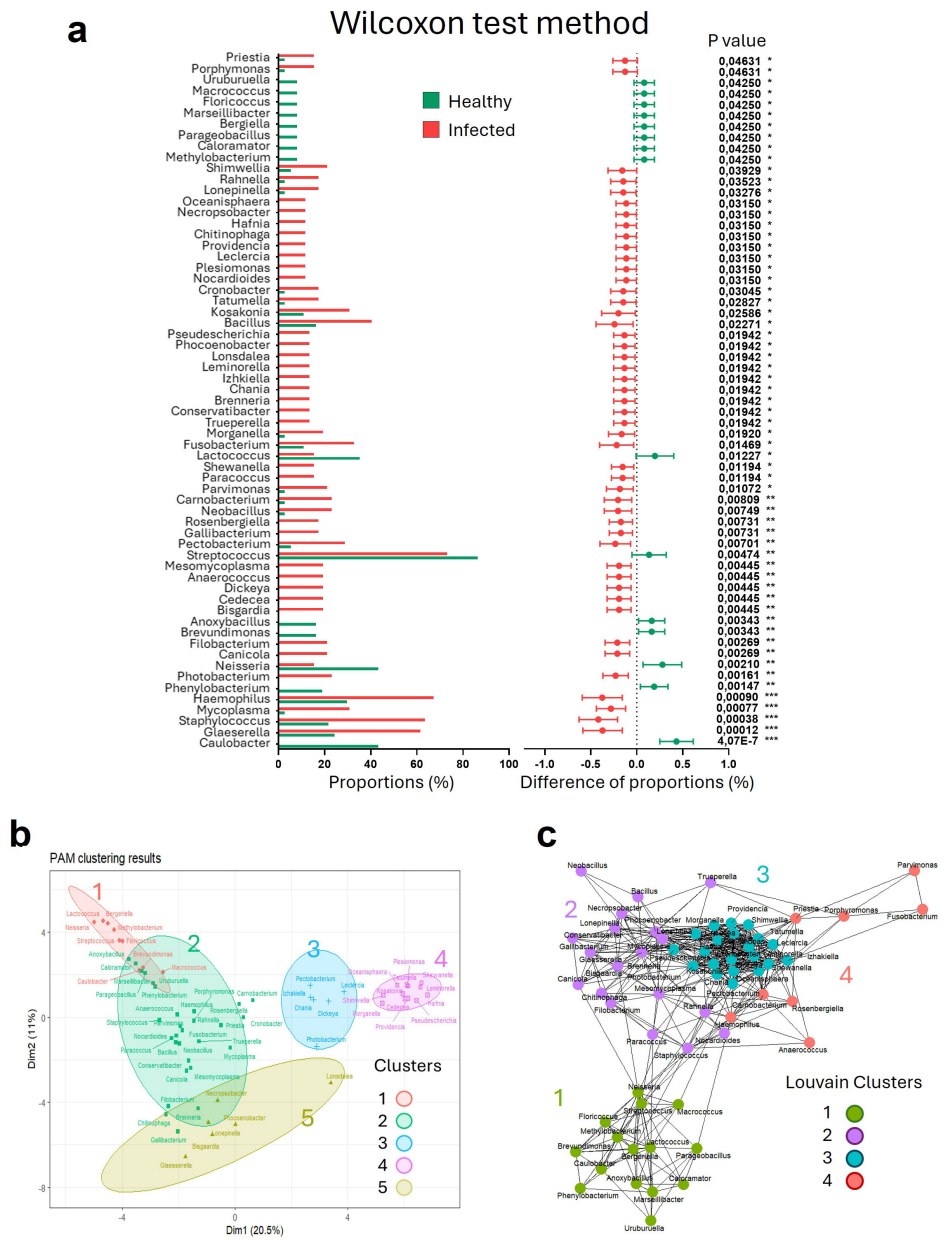
Differential bacterial proportions and correlation networks. **(a)** Comparisons of the relative proportion (%) in Influenza-virus infected and healthy groups at the level of bacterial genus. **(b)** Partitioning Around Medoids (PAM) clustering analysis. **(c)** Neural network of significant bacteria based on Pearson correlation and Louvain clusterization. Each node for **(b, c)** represents a bacterial genus, separated by distances and grouped following clusterization methods.

The Wilcoxon rank-sum test revealed significant differences in the abundance of 63 bacterial taxa between the infected and healthy groups (**Figure 3a**). Of these, 14 taxa were predominantly associated with the healthy group, showing higher prevalence and mean abundances, while the remaining taxa were more indicative of the infected group. Bacterial group from healthy samples included *Caulobacter, Phenylobacterium, Neisseria, Brevundimonas, Anoxybacillus, Streptococcus* and *Lactococcus*, among others. The complete list of significantly different taxa, including those associated with the infected group and their corresponding p-values, is fully reported in **Figure 3a**.

Using only the 63 significant bacteria, the clustering method based on *k*-medoids (Partitioning Around Medoids – PAM) differences between 5 clusters of bacteria, of which healthy samples are embedded in cluster 1 area (**Figure 3b**). However, neural network based on Louvain communities of Pearson correlation showed 4 optimal clusters. Here, cluster 1 shows the interaction between all significant bacterial species for healthy samples (**Figure 3c**). The other 3 clusters, corresponding to bacteria predominantly found in infected samples, cluster together and are separated from cluster 1. Although no single ‘primary’ bacterium dominates each cluster, several taxa within each group stand out either by their high abundance or clinical relevance. For example, *Glaesserella* or *Fusobacterium* were prominent in clusters associated with infected animals, both known respiratory pathogens in pigs. In healthy groups, *Streptococcus, Neisseria*, or *Lactococcus.*

## Discussion

Since the relationship between lung microbiota and respiratory infections was first explored, several studies have focused on describing bacteria present in the lungs of infected animals, including those infected with Influenza viruses (Vereecke et al., 2023). Human studies, for instance, have analyzed bronchoalveolar lavages from critically ill patients with community-acquired pneumonia (CAP) (Marimón et al., 2023; Zhou et al., 2023a). However, these studies are often limited by small sample sizes, biased due to preventive antibiotic treatments, and cross-contamination from the mouth microbiota, among other variables. Increasing the number of samples and conducting broader studies pose significant challenges when analyzing human samples. This issue, however, can be mitigated by using swine samples, as the lung structure and immune response in swine closely mimic those in humans (Schwaiger et al., 2019).

To date, no published studies have investigated the swine lung-tissue microbiota in the context of Influenza virus infection. Some of them, instead focused on lung microbiota of diseased pigs (Porcine Respiratory Disease Complex – PRDC) (Li et al., 2021), or studied the use of probiotics to prevent pathology in the lung microbiota composition (Winther et al., 2024). Others characterized the swine lower respiratory tract antibiotic resistome (Zhou et al., 2023b). A recent similar study identified viral and bacterial profiles in endemic Influenza A virus infected swine herds using Nanopore metagenomic sequencing on tracheobronchial swabs (Vereecke et al., 2023).

On the other hand, although human and swine microbiotas are different in the lungs (Yu et al., 2016; Siqueira et al., 2017), it is critical to understand swine microbiota alterations associated with Influenza virus and lung microbiota dynamics, because pigs are anatomically and physiologically similar to humans (Rajao and Vincent, 2015). Consequently, the swine model could be potentially used to explore the understanding the potential source of inflammatory signals and potential future therapeutic approaches associated with influenza complications. For this reason, we analyzed the pulmonary microbiota of 53 pigs infected with Influenza and compared them with the microbiota of 39 healthy pigs. All samples belonged to different geographic areas from Spain. However, as a limitation, no detailed clinical data was available beyond the general symptomatology and influenza positivity. Thus, we cannot correlate disease severity with bacterial coinfections or metagenomic findings. Notably, defining the clinical course of influenza in pigs, including any signs of severe respiratory distress, could aid in identifying coinfection markers for more comprehensive future analyses.

Bacterial communities in lung samples Influenza-infected and non-infected animals present significant differences in terms of detection (presence) and relative abundance. The bacterial 16S/18S DNA ratio can be used as a surrogate of the relative abundance of bacteria in a lung sample as compared to eukaryotic cells. The analysis of this ratio by qPCR indicates an overall increase of bacterial 16S compared to 18S in Influenza-infected samples (**Figure 2a**) that can correlate with a higher abundance of bacterial communities in infected samples (**Supplementary Figure 1b**).

Although all samples were manipulated with the same DNA extraction protocols, there are important differences in the DNA quality that could be attributed to differences in sample collection, processing, and preservation, as they were collected at different origins and time points. Also, the distribution of bacteria in the lung might differ among pigs (Dickson et al., 2015a). Despite that, clear differences can be observed in infected samples.

Initial analysis of bacterial 16S sequencing indicates that alpha diversity showed no significant differences (**Supplementary Figure 1a**), although the rarefaction curves (**Supplementary Figure 1b**) showed higher bacterial richness in infected animals. In line with this observation, other microbiome articles in influenza-infected humans showed no significance in alpha diversity neither (Zhou et al., 2023a). A possible explanation for this phenomenon could be that diversity indexes consider not only the richness of genera, but also the distribution of those species. The samples from infected animals revealed increased richness, yet a higher imbalance in genus abundance, resulting in the dominance of particular bacterial species. This aligns with the scenarios described by Jake G. Natalini et al. (Natalini et al., 2023), where dysbiosis can increase host susceptibility to pathogens. The authors explain that dysbiosis may favor dominant bacteria in the lungs, or, in their absence, promote the growth of less dominant pathogens, leading to further dysbiosis and pathogenesis.

On the other hand, principal coordinates analysis (PCoA) of Bray-Curtis (BC) distances showed some differences between the two groups. However, the percentage of explanation from the two first dimensions are low (9.34% and 7.85%, respectively). This could be explained by the wide variability of the samples in terms of origin, timepoint of collection, and other factors such as the Influenza virus subtype causing the infection or divergent swine genetics. The 3-dimensional MDS plot shown in **Supplementary Figure 1d**, which captures an additional 6.18% of the variance between samples, illustrates a distinct clustering of the healthy samples (shown in green) near the center. In contrast, the infected samples are dispersed radially in multiple directions, rather than deviating along a single axis, suggesting a widespread divergence from the healthy group in various aspects of microbial composition. Finally, in terms of potential biases stemming from batch effects or sample origin, no clear evidence was found indicating any significant influence on the results of the samples.

Mean relative abundance analysis indicates that Influenza-infected animals present a significantly higher abundance of potential pathogenic bacteria, responsible for the majority of secondary infections in pigs (Obradovic et al., 2021; Goto et al., 2023). In particular, *Glaesserella* spp. was found approximately in 60% of our infected samples and, in some cases, was found to be the most predominant bacteria in those samples. Other bacteria, such as *Pasteurella*, *Staphylococcus, Mycoplasma* or *Fusobacterium* were also correlated with infected samples. These bacterial genres are common secondary pathogens in swine respiratory disease (SRD) (Goto et al., 2023). In particular, *Glaesserella parasuis*, *Pasteurella multocida, and Mycoplasma hyopneumoniae* are responsible for the porcine respiratory disease complex (PRDC) along with Influenza virus (Zimmerman et al., 2019). These bacteria correlated significantly with infected samples after performing statistical analysis by Wilcoxon (**Figure 3a**). Then, although the Porcine Reproductive and Respiratory Syndrome (PRRS) is commonly associated with Influenza virus and *Actinobacillus* spp, we found no significant differences in the detection of this bacteria in healthy or infected samples.

Conversely, certain bacterial genera were significantly correlated specifically with the healthy group, although they were fewer in comparison to the samples from Influenza-infected animals. Among them, *Streptococcus* spp.*, Caulobacter* spp., and *Lactococcus* spp. were notably associated with samples from uninfected animals (**Figure 3a**). Some of these bacteria, such as *Lactococcus*, have been repeatedly associated with higher prevalence and abundance in healthy samples when compared to infected ones. For instance, in one study of the microbiota of diseased pigs with porcine respiratory disease complex (PRDC) (Li et al., 2021). Others, like *Streptococcus* spp., are part of the normal pulmonary microbiota, as they are known to colonize the respiratory tract, likely due to their common presence in the oral cavity, which allows direct contact with the lungs (Obradovic et al., 2021).

In particular with *Streptococcus*, we observed high prevalence in both healthy (84%) and infected (72%) samples, as can be observed in **Supplementary Figure 2**. However, the differences observed between healthy and infected samples are not solely based on genus-level abundances, but also on species-level distinctions. Although we cannot confirm this data due to 16S sequencing limitations on taxonomic resolution (Benítez-Páez et al., 2016), initial 16S-based identification indicated that infected samples exhibit a higher prevalence of specific *Streptococcus* species, such as *S. suis*, the most common, as well as *S. porcinus* and *S. porci*. In contrast, healthy samples, while also harboring these colonizing species, show a greater diversity of *Streptococcus*, including additional species like *S. agalactiae*, *S. pyogenes*, and *S. parasuis*, among others.

Finally, we used two clustering methods to determine the relationship between genera in the samples (**Figures 3b, c**). The first method, PAM, partitions the samples into groups by minimizing the sum of dissimilarities between points and their assigned medoids. This allows for the possibility that a bacterium, while assigned to one group, may be closer to the medoid of another, suggesting that it could potentially belong to more than one group. On the other hand, the Louvain method identifies communities based on the maximization of modularity in a network, with interactions between nodes (bacteria), though each node can only belong to a single group or community.

The Louvain clustering method for community detection identified four distinct groups, of which one of them (Cluster 1 – **Figure 3c**) was separated, corresponding to bacteria that are more characteristic of healthy samples. Additionally, the subclustering from what we considered the three infection-associated groups, may indicate three potential factors: (i) variability within the infected group due to differences in the pre-infection commensal microbiota of the pigs, possibly influenced by factors such as environmental factors, animal age, disease progression, immune response, or prior treatments; (ii) the presence of distinct subpopulations of bacteria within infected samples derived from the infection stage, severity, or coinfections, potentially reflecting differences in microbial composition; and (iii) the interactions among various bacterial species, with some acting as primary pathogens, while others function as secondary bacteria, taking advantage of the environment created by the primary bacterial invasion. Additionally, the subtype of Influenza virus infecting the pigs may play a role in this subclustering. Different Influenza subtypes could influence the pulmonary microbiota in distinct ways, potentially leading to variations in bacterial community structure. More virulent subtypes, or more serious disease, might disrupt the microbiota more severely, allowing specific pathogens to dominate, which could explain the observed differences between the subclusters. In fact, we identified multiple influenza subtypes across our samples. However, the subtyping analysis was not conducted due to incomplete data availability for all pigs. These aspects should be clarified in future analysis.

While this study provides valuable insights into the pulmonary microbiota of naturally influenza-infected farm pigs, several limitations should be addressed. One major limitation is the low bacterial yield from several lung samples, which increases the potential for bias. Although we mitigated this issue by including a relatively large sample size (92 samples), a more robust and statistically representative analysis would require an even larger number of samples from more diverse environments. Furthermore, the lack of detailed information about the pigs, such as any treatments they may have received, the timing of lung sample collection relative to the onset of symptoms, or the exact time since infection, constrains our ability to fully understand the factors driving the observed microbial shifts. These unknown variables introduce potential confounders that could influence bacterial composition. Due to confidentiality agreements with the farms involved in the study, we were not granted access to detailed metadata of the animals, such as sex, age, breed, reproductive status, diet, or medical history. While these variables can influence the microbiota and would have enriched the interpretation of our findings, their unavailability represents an inherent limitation of working with field samples under real-world conditions. A further limitation is the potential variability in sample collection. Although biopsies were generally taken from comparable lung regions (preferably the distal right cardiac lobe) and inflamed areas in infected animals, sampling was performed by different personnel across multiple sites without centralized supervision. This may have introduced variability in the microbial profiles, which should be considered when interpreting the results.

Despite these limitations, our study has notable strengths. It is among the firsts to explore the pulmonary microbiota in farm pigs naturally infected with circulating strains of Influenza, offering a comparison with healthy pigs from farm environments. Unlike laboratory studies, which often operate under highly controlled conditions where the microbiota, diet, and environment are more homogeneous, our study captures the complex and variable realities of commercial farming. In laboratory settings, environmental factors are tightly regulated, which can facilitate analysis but may not accurately reflect real-world conditions, as farm environments are typically more diverse and dynamic and can potentially be less clean. This makes our findings particularly relevant for understanding microbial dynamics in pigs under natural farming conditions, where external influences such as diet, housing, and hygiene are less controlled but more representative of the actual conditions faced in swine production systems.

In this sense, we also tested lung samples from controlled *in vivo* experiments performed in an animal housing facility (data not shown). In our case, the number of individual species obtained was very limited compared to the farm animals. We attribute this low number of species to the animal housing conditions, which are normally clean and more homogenous compared to farms. This might limit the use of *in vivo* models to study pigs’ microbiota associated with influenza.

Although studies in humans are limited by the difficulty of accessing lung tissue directly, investigations using sputum or bronchoalveolar lavage samples have reported changes in lung microbiota associated with influenza A infection. For instance, a recent study found significant shifts in the abundance of genera such as *Neisseria*, *Porphyromonas*, and *Actinobacillus* in patients with severe influenza A pneumonia, despite no significant differences in overall diversity metrics (Zhou et al., 2023a). These findings support the notion that influenza infection impacts lung bacterial communities, highlighting the translational relevance of our pig lung tissue study, which benefits from direct sampling of lung parenchyma and controlled experimental conditions.

The complex inflammatory regulation of the host-respiratory and immune systems during influenza determines the severity of the disease. While much attention has been given to individual pathogens, such as different Influenza viruses and the bacteria associated with secondary infections, this traditional view may be overly simplistic. The data suggest that Koch’s postulates no longer fully explain the complexity of the disease caused by some infectious agents. The role of different bacteria associated with infections by Influenza viruses requires further investigation. Alternative dysbiosis contexts can lead to similar disease severities, requiring different therapeutic approaches. Both descriptive and functional microbiota assays are essential to better understand disease etiology and develop more effective treatments.

## Data Availability

The raw sequencing data used and described in this study have been deposited into both National Genomics Data Center (NGDC) (https://ngdc.cncb.ac.cn/) of China National GeneBank DataBase (CNGBdb) with accession number PRJCA032289, and in the NCBI Sequence Read Archive (SRA) with accession number PRJNA1285568.
