# Supplementary material for: Swine influenza-modified pulmonary microbiota
Source: Front Cell Infect Microbiol. 2025 Sep 8;15:1634469. doi: 10.3389/fcimb.2025.1634469 (PMC12450909; doi:10.3389/fcimb.2025.1634469)
Supplement: Supplementary file 1 [file DataSheet1.pdf]

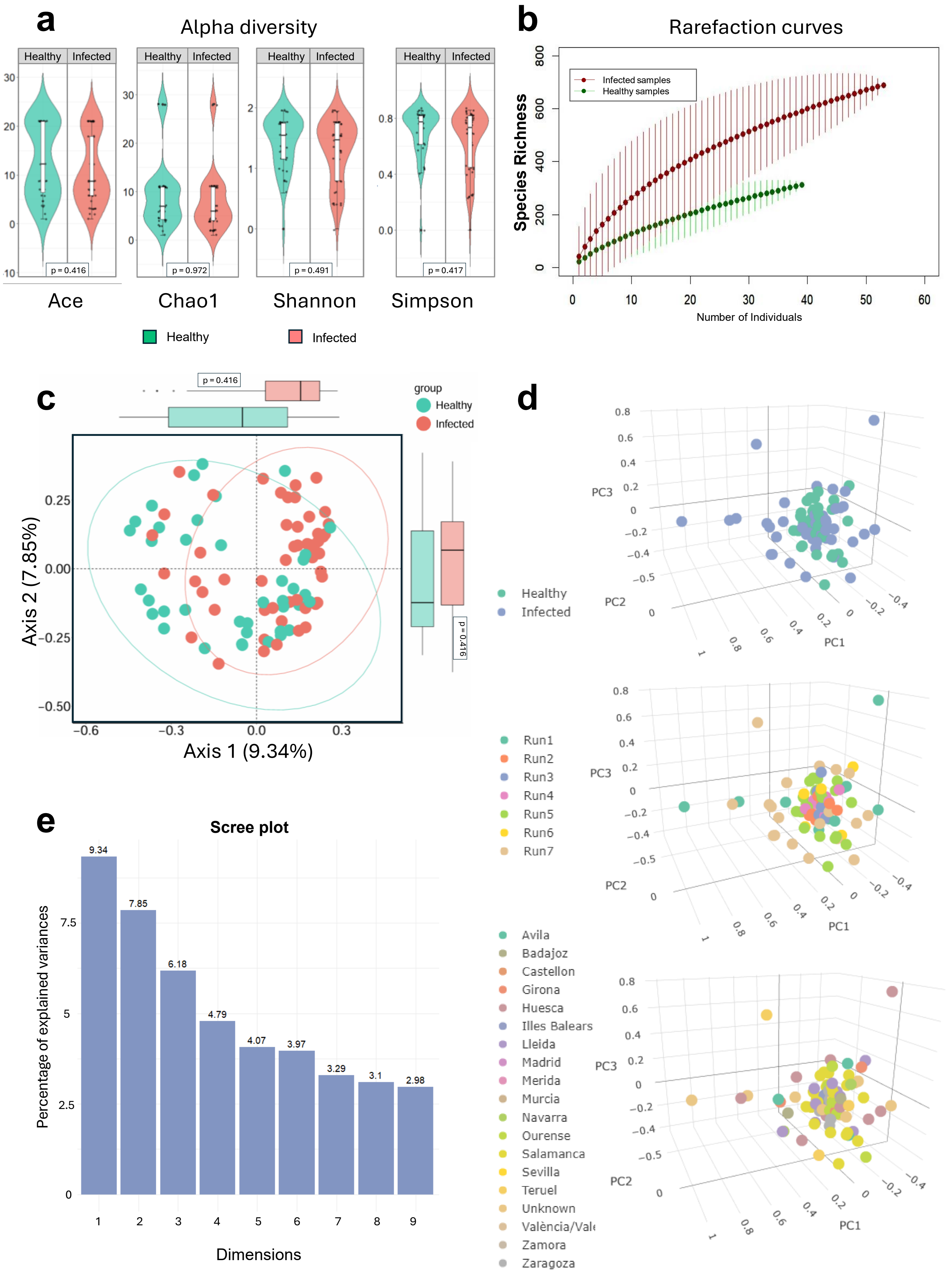
a
Alpha diversity
Healthy
Infected
p = 0.416
p = 0.972
p = 0.491
p = 0.417
Ace
Chao1
Shannon
Simpson
b
Rarefaction curves
Infected samples
Healthy samples
Species Richness
Number of Individuals
c
group
Healthy
Infected
p = 0.416
Axis 2 (7.85%)
Axis 1 (9.34%)
d
PC1
PC2
PC3
Run1
Run2
Run3
Run4
Run5
Run6
Run7
Avila
Badajoz
Castellon
Girona
Huesca
Illes Balears
Lleida
Madrid
Merida
Murcia
Navarra
Ourense
Salamanca
Sevilla
Teruel
Unknown
València/Vale
Zamora
Zaragoza
e
Scree plot
Percentage of explained variances
Dimensions
9.34
7.85
6.18
4.79
4.07
3.97
3.29
3.1
2.98

a

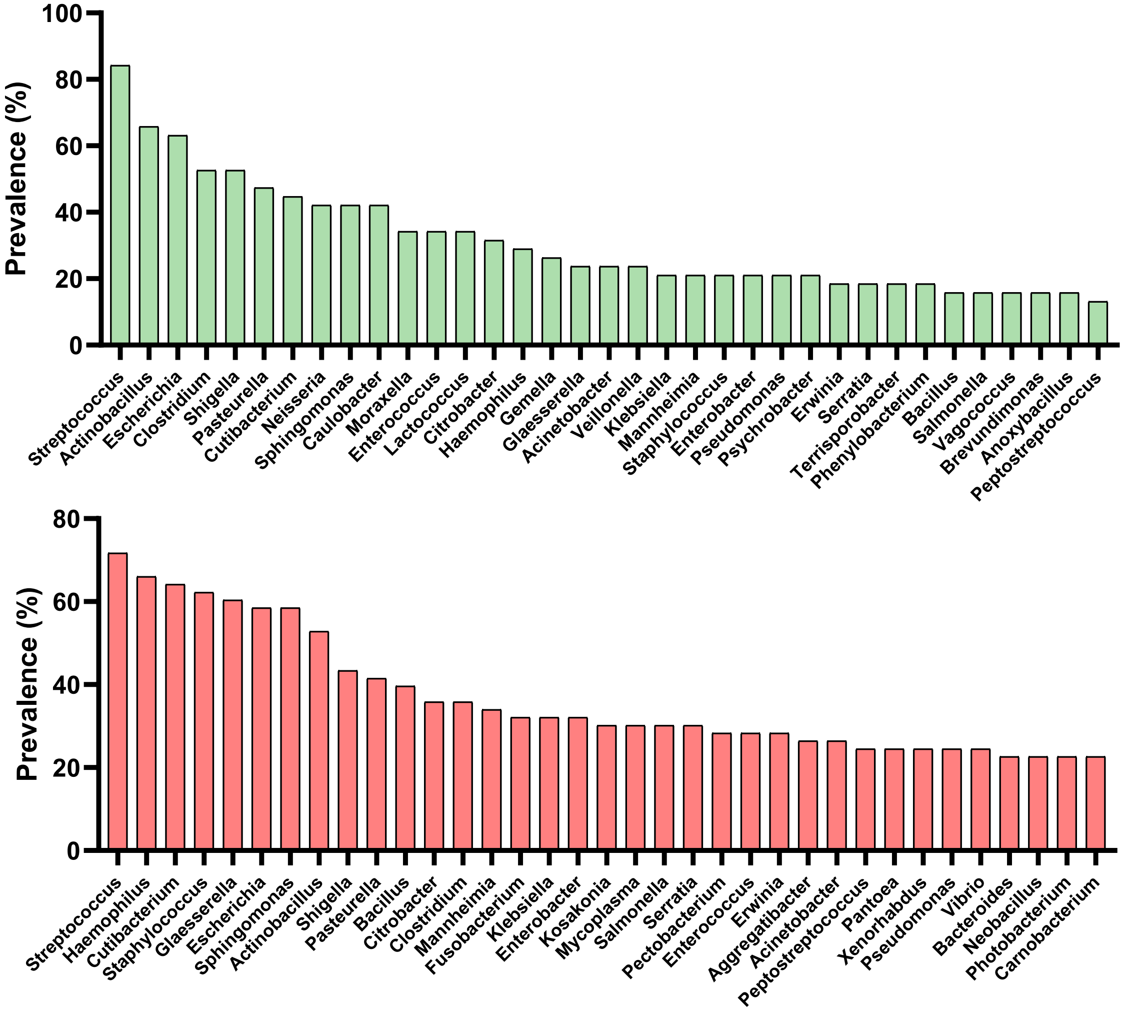

b

Healthy		Both			Infected	
**Genus**	**Prevalence Healthy (%)**	**Genus**	**Prevalence Healthy (%)**	**Prevalence Infected (%)**	**Genus**	**Prevalence Infected (%)**
Caulobacter	42%	Streptococcus	84%	72%	Photobacterium	23%
Phenylobacterium	18%	Escherichia	63%	58%	Canicola	21%
Brevundimonas	16%	Actinobacillus	66%	53%	Filobacterium	21%
Anoxybacillus	16%	Cutibacterium	45%	64%	Bisgaardia	19%
Methylobacterium	08%	Sphingomonas	42%	58%	Cedecea	19%
Caloramator	08%	Haemophilus	29%	66%	Dickeya	19%
Parageobacillus	08%	Shigella	53%	43%	Anaerococcus	19%
Bergeriella	08%	Glaesserella	24%	60%	Mesomycoplasma	19%
Marseillibacter	08%	Staphylococcus	21%	62%	Gallibacterium	17%
Floricoccus	08%	Pasteurella	47%	42%	Rosenbergiella	17%
Macrococcus	08%	Clostridium	53%	36%	Shewanella	15%
Uruburuella	08%	Citrobacter	32%	36%	Paracoccus	15%
Chelatococcus	05%	Enterococcus	34%	28%	Trueperella	13%
Janthinobacterium	05%	Bacillus	16%	40%	Conservatibacter	13%
Acidithiobacillus	05%	Mannheimia	21%	34%	Brenneria	13%
Croceibacterium	05%	Klebsiella	21%	32%	Chania	13%
Fermentimonas	05%	Enterobacter	21%	32%	Izhakiella	13%
Hydrogenophilus	05%	Neisseria	42%	15%	Leminorella	13%
Proteiniphilum	05%	Moraxella	34%	21%	Lonsdalea	13%
Tidjanibacter	05%	Acinetobacter	24%	26%	Phocoenobacter	13%

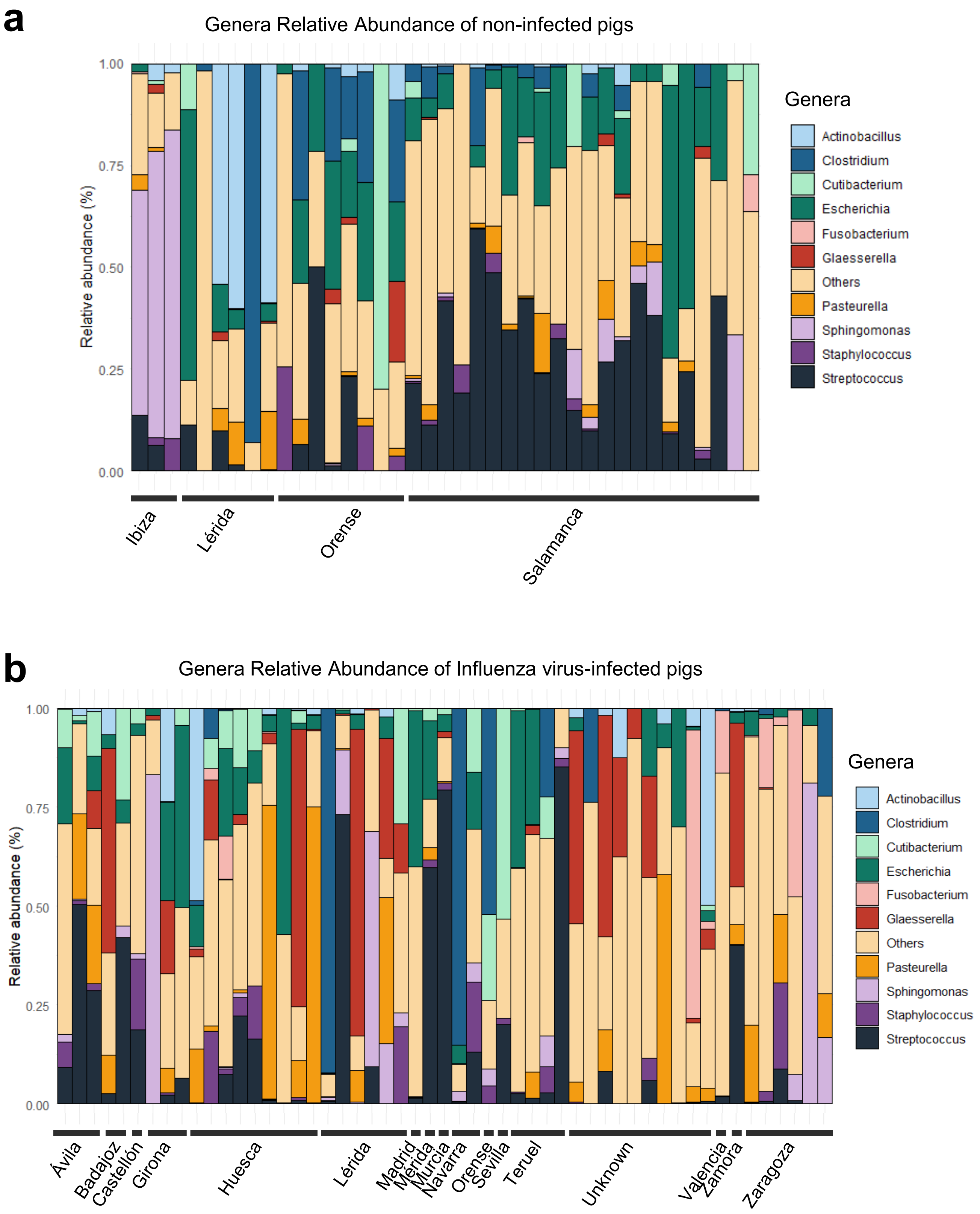
a
Genera Relative Abundance of non-infected pigs
Relative abundance (%)
1.00
0.75
0.50
0.25
0.00
Ibiza
Lérida
Orense
Salamanca
Genera
Actinobacillus
Clostridium
Cutibacterium
Escherichia
Fusobacterium
Glaesserella
Others
Pasteurella
Sphingomonas
Staphylococcus
Streptococcus
b
Genera Relative Abundance of Influenza virus-infected pigs
Relative abundance (%)
1.00
0.75
0.50
0.25
0.00
Ávila
Badajoz
Castellón
Girona
Huesca
Lérida
Madrid
Mérida
Murcia
Navarra
Orense
Sevilla
Teruel
Unknown
Valencia
Zamora
Zaragoza
Genera
Actinobacillus
Clostridium
Cutibacterium
Escherichia
Fusobacterium
Glaesserella
Others
Pasteurella
Sphingomonas
Staphylococcus
Streptococcus

a

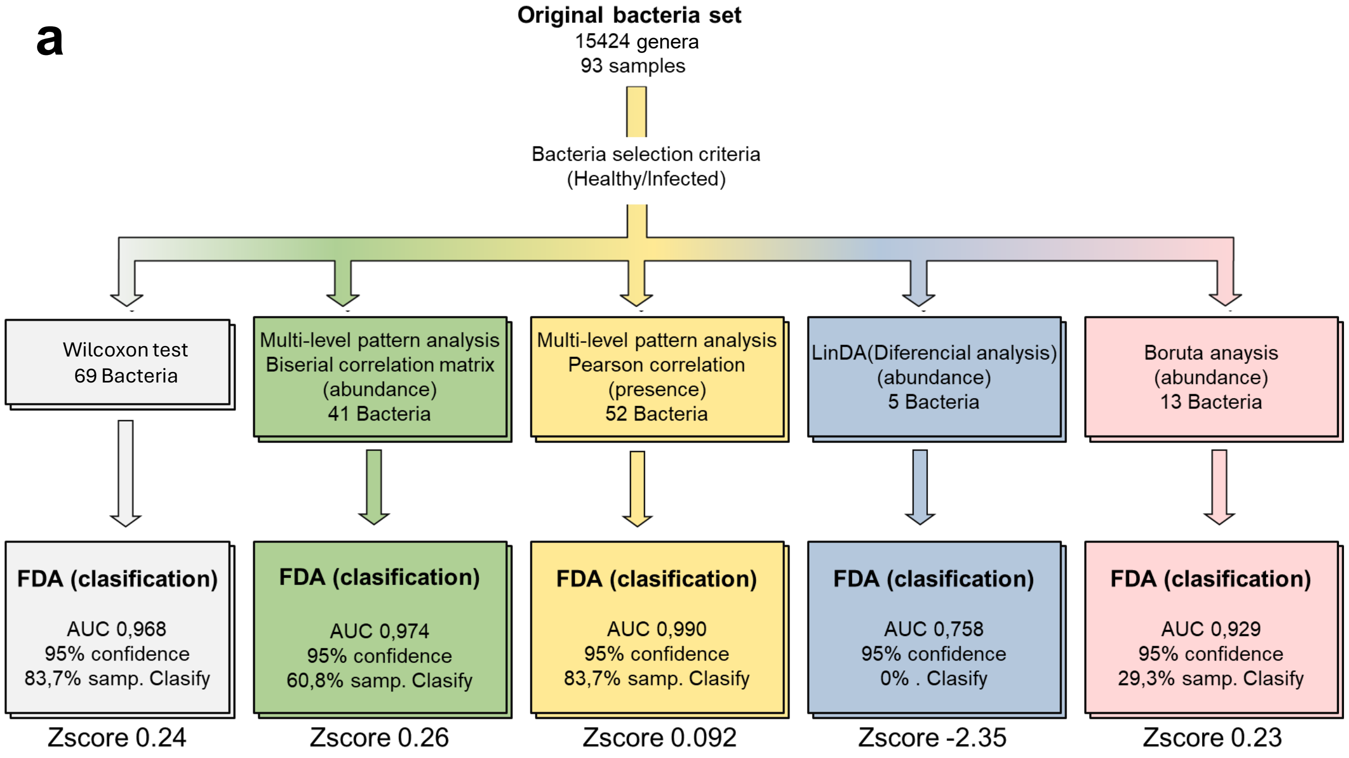

b

Method	Description	Data type	Significant Genera ($p < 0.05$)	Sample Classification (%)	Zscore
**Wilcoxon rank-sum test**	Non-parametric test that compares the distribution of relative abundances between two groups	Relative abundance	69	83.7	0.24
**Biserial correlation (multipatt)**	Computes the correlation between genus presence/absence and group membership	Presence/absence	41	60.8	0.26
**Pearson with presence/absence (multipatt)**	Calculates linear correlation between genus presence and group membership	Presence/absence	52	83.7	0.092
**LinDA (microbiomeStat)**	Linear model adjusted for confounders to detect differential abundance	Adjusted abundance	5	0	-2.35
**Boruta**	Random Forest-based method to identify genera with predictive value	Relative abundance	13	29.3	0.23

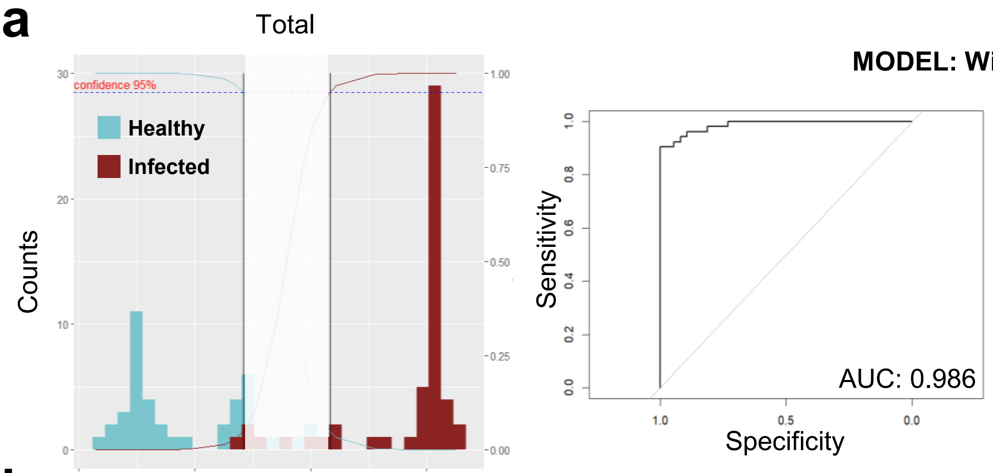

Total

Predicted	Healthy	Infected
Healthy	35	5
Infected	2	47

>95% confindence

Predicted	Healthy	Infected
Healthy	30	2
Infected	0	45

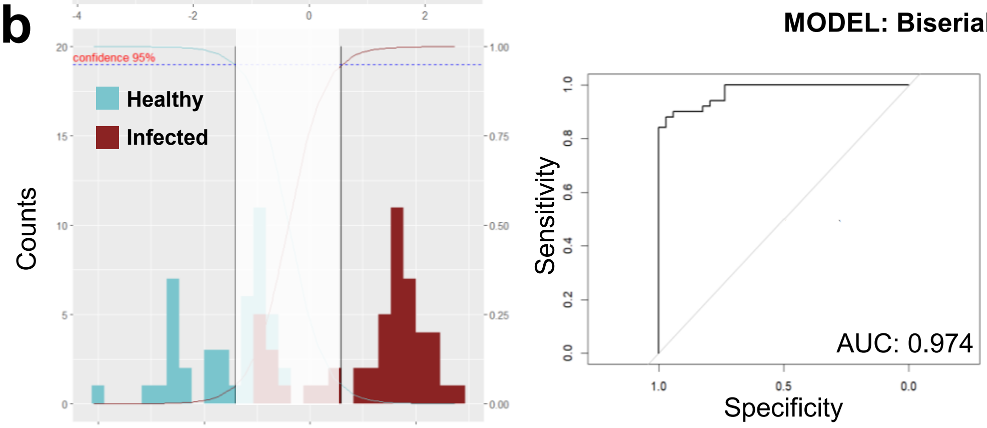

Total

Predicted	Healthy	Infected
Healthy	34	9
Infected	0	41

>95% confindence

Predicted	Healthy	Infected
Healthy	19	0
Infected	0	37

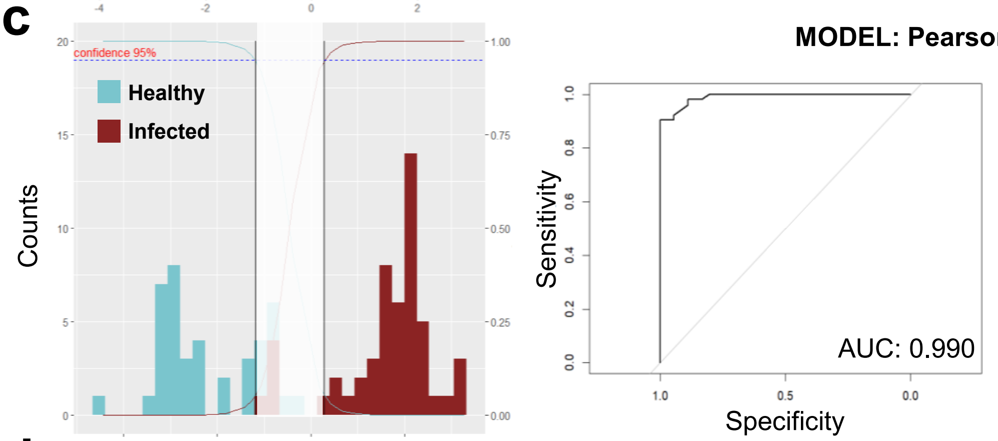

Total

Predicted	Healthy	Infected
Healthy	35	5
Infected	1	47

>95% confindence

Predicted	Healthy	Infected
Healthy	30	1
Infected	0	46

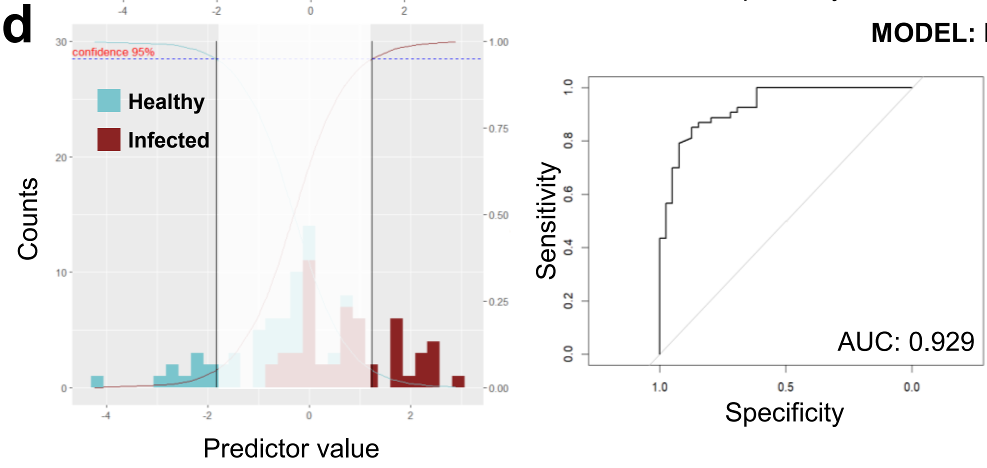

Total

Predicted	Healthy	Infected
Healthy	28	6
Infected	11	47

>95% confindence

Predicted	Healthy	Infected
Healthy	10	0
Infected	0	17
